# IKAROS regulates human T cell phenotype at a thymic and postthymic level

**DOI:** 10.1172/jci.insight.197359

**Published:** 2025-12-22

**Authors:** Jennifer Stoddard, Hye Sun Kuehn, Ravichandra Tagirasa, Marita Bosticardo, Francesca Pala, Julie E. Niemela, Agustin A. Gil Silva, Kayla Amini, Eduardo Anaya, Mario Framil Seoane, Carolina Bouso, Dimana Dimitrova, Jennifer A. Kanakry, Laia Alsina, Matias Oleastro, Steven M. Holland, Thomas A. Fleisher, Richard L. Wasserman, Luigi D. Notarangelo, Sergio D. Rosenzweig

**Affiliations:** 1 Immunology Service, Department of Laboratory Medicine, Clinical Center, NIH, Bethesda, Maryland, USA.; 2 Laboratory of Clinical Immunology and Microbiology, National Institute of Allergy and Infectious Diseases, NIH, Bethesda, Maryland, USA.; 3 Immunology Department, Hospital Clínic, Barcelona, Spain.; 4 Department of Immunology and Rheumatology, Juan P. Garrahan National Pediatric Hospital, Buenos Aires, Argentina.; 5 Center for Immuno-Oncology, National Cancer Institute, NIH, Bethesda, Maryland, USA.; 6 Study Group for Immune Disfunction Diseases in Children, Institut de Recerca Sant Joan de Déu, Clinical Immunology and Primary Immunodeficiencies Unit, Pediatric Allergy and Clinical Immunology Department, Hospital Sant Joan de Déu, Barcelona, Spain.; 7 Department of Pediatrics, Medical City Children’s Hospital, Dallas, Texas, USA.

**Keywords:** Cell biology, Genetics, Immunology, T cells

## Abstract

The transcription factor IKAROS, encoded by *IKZF1*, is crucial for lymphocyte development and differentiation. Germline heterozygous *IKZF1* mutations cause B cell immunodeficiency, but also affect T cells. Patients with *IKZF1* haploinsufficiency (HI) or dimerization-defective (DD) variants show reduced naive and increased memory T cells, while dominant-negative (DN) mutations result in the opposite phenotype. Gain-of-function patients display variable patterns. To investigate IKAROS’s role in shaping the human naive/memory T cell phenotype, we performed IKAROS immunomodulation and knockdown experiments and analyzed early T cell development in an artificial thymic organoid (ATO) system using CD34^+^ cells from patients with representative *IKZF1* variants. IKAROS inhibition by lenalidomide or silencing by small hairpin RNA directly altered expression of HNRNPLL, the master regulator of CD45 isoform splicing that defines CD45RA^+^/naive and CD45RO^+^/memory phenotypes. In the ATO system, IKAROS-DN precursor cells were blocked at the CD4^–^CD8^–^/double-negative stage and retained a CD45RA^+^ phenotype, whereas IKAROS-HI cells inefficiently reached the CD4^+^CD8^+^/double-positive stage and partially transitioned from CD45RA to CD45RO. Analysis of public gene expression data showed high HNRNPLL expression in double-positive thymic cells, beyond the stages affected by *IKZF1* DN and HI mutations. Collectively, these findings indicate that IKAROS regulates early and late T cell development by mechanisms, including HNRNPLL modulation.

## Abstract 2

IKAROS, a transcription factor critical for lymphocyte development, differentiation and a tumor suppresor, regulates human T-cell phenotype and funtion at a thymic and post-thymic level.

## Introduction

The transcription factor IKAROS, encoded by the *IKZF1* gene, is a master regulator of lymphocyte development and hematopoiesis as well as a tumor suppressor (1). IKAROS contains an N-terminal DNA binding domain made up of 4 zinc fingers (ZF1–4) and a C-terminal dimerization domain with 2 zinc fingers (ZF5–6). IKAROS mainly functions as a gene repressor by binding to DNA at the pericentromeric heterochromatin regions and by interacting with the polycomb repressive and chromatin remodeling and deacetylase complexes (1, 2). In humans, deleterious heterozygous germline mutations in *IKZF1* lead to primary immunodeficiency/inborn errors of immunity with increased susceptibility to infections, immune dysregulation, and malignancies (3–5). *IKZF1* mutations are classified into 4 main groups based on their mechanism of action and functional effects: haploinsufficiency (HI; DNA binding), dimerization defective (DD; dimerization), dominant negative (DN; DNA binding), and gain of function (GOF; DNA binding) (3). While the B cell compartment is systematically affected across all of the above-mentioned allelic variants (3), the T and myeloid lineages are variably impacted. This is particularly evident among patients carrying DN mutations, presenting with severe B cell lymphopenia, as well as neutropenia, eosinopenia, and an unusual T cell phenotype, characterized by sustained CD45RA and CD31 expression, with limited cytokine production and Th polarization, altogether resembling naive, recent thymic emigrant, Th0 cells. Even upon in vitro stimulation, these cells show a very limited capacity to mature or modify their immunophenotype (6). In T cell biology, the transition from a CD45RA^+^ naive to a CD45RO^+^ memory phenotype is characterized by the splicing-dependent switching of the high molecular transmembrane phosphatase CD45RA isoform to lower-molecular-weight variants, including CD45RO (7). Activation of T cells induces the expression of HNRNPLL, an RNA-binding tyrosine phosphatase and master regulator of *PTPRC/CD45* splicing, underscoring the critical role of HNRNPLL in T cell development, activation, and function (8).

Herein, we investigated the role of IKAROS in T cell phenotype modulation, CD45 isoform expression, and HNRNPLL regulation using in vitro immunomodulatory treatment and knockdown studies on healthy control naive CD4^+^ T cells. We also investigated to what extent *IKZF1* variants exerting DN versus HI effects impinge on in vitro T cell development and the transition from CD45RA to CD45RO expression in an artificial thymic organoid (ATO) system. Altogether, these data provide insights into the thymic and peripheral mechanisms through which IKAROS affects T cell development.

## Results

### T cell immunophenotype in patients with IKAROS-associated diseases.

**Figure 1:**
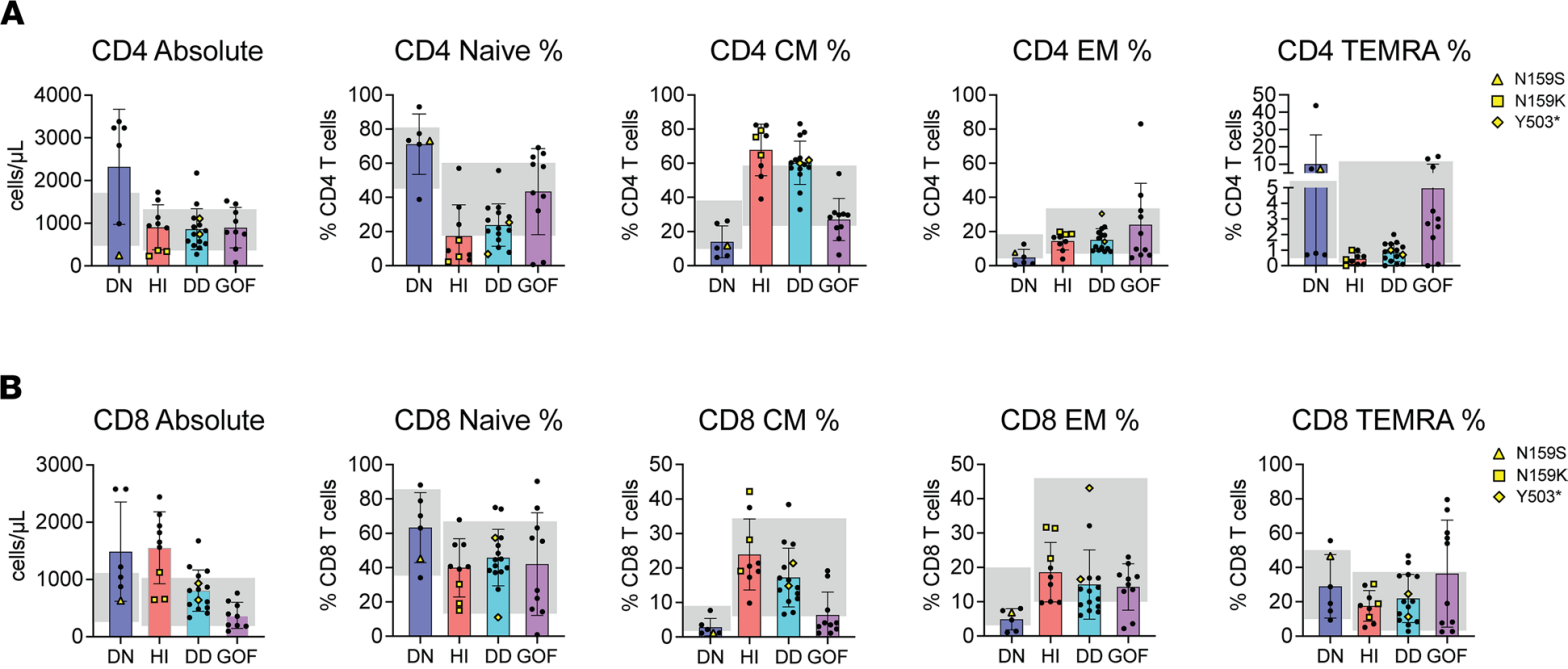
Naive and memory T cell phenotypes in patients with IKZF1 mutations. (**A**) Naive CD4^+^ T (CD45 RA^+^CD62L^+^), central memory (CM; CD45RA^–^CD62L^+^), effector memory (EM; CD45RA^–^CD62L^–^), and TEMRA (CD45RA^+^CD62L^–^) cells are shown. Each dot represents a different individual. DN (total *n* = 6): IKZF1^N159S^ (*n* = 5) (6), IKZF1^N159T^ (*n* = 1) (6); HI (total *n* = 9): IKZF1^N159K^ (*n* = 3), IKZF1^H167R^ (*n* = 3) (9), IKZF1^R162L^ (*n* = 2) (9), IKZF1^R184Q^ (*n* = 1) (9); DD (total *n* = 15) IKZF1^Y503*^ (*n* = 4) (13), IKZF1^R213*^ (*n* = 2) (11), IKZF1^S427*^ (*n* = 1) (11), IKZF1^C467R^ (*n* = 6) (11), IKZF1^R502L^ (*n* = 2) (11); and GOF (total *n* = 10): IKZF1^R183H^ (*n* = 5) (10), IKZF1^R183C^ (*n* = 3) (10), IKZF1^T398M^ (*n* = 2) (12) (**A**-**B**) CD4+ T cell (**A**) and CD8+ T cell (**B**) data are shown. Naive (CD45 RA+CD62L+), central memory. Black circles indicate values from previously reported patients, and yellow symbols represent newly identified, previously unpublished patients included in this study. Shaded area in graphs represent healthy control normal ranges (*n* = 40, 95% CI) from pediatric (DN) and adult (HI, DD, GOF) populations. Data are represented as mean ± SD.

As the number of patients with IKAROS-associated diseases continues to grow, distinctive T cell immunophenotypic patterns are becoming readily identifiable in patients carrying DN, HI, DD, and GOF mutations (6, 9–13). In a primary T cell immunophenotyping analysis, we included previously published cases (6, 9–13) (represented by full circles in Figure 1) as well as new patients and mutations tested for this work (represented by yellow rectangles, diamonds, and triangles) (Supplemental Figure 1 and Supplemental Table 1; supplemental material available online with this article; https://doi.org/10.1172/jci.insight.197359DS1). When absolute and percentual CD4^+^ T cell subsets were analyzed (i.e., naive, central memory [CM], effector memory [EM], and EM reexpressing RA [TEMRA] cells) and compared across IKAROS allelic variants and individuals acting as healthy controls (HCs), a distinctive pattern became evident. DN and GOF patients displayed increased naive and TEMRA/decreased CM cells, while patients with HI and DD allelic variants presented the opposite picture, with increased CM/decreased naive and TEMRA cells (Figure 1A and Supplemental Figure 2). Analysis of CD8^+^ T cells followed a similar, although less pronounced, trend as CD4^+^ T cells both at absolute and percentual values (Figure 1B).

Among the newly tested patients/variants for this work, we investigated a family of 3 individuals, including a father and his 2 children, carrying what we believe to be a previously unreported variant, N159K, and presenting with a common variable immunodeficiency–like (CVID-like) phenotype. IKAROS functional assays, including immunofluorescence and EMSA, indicated that the N159K variant is a loss-of-function (LOF) mutation, as it completely fails DNA binding. Furthermore, cotransfection studies showed no DN effect on pericentromeric targeting, demonstrating that the mutant behaved in a HI manner. (Supplemental Figure 3). It is noteworthy that missense variants at position 159 (N159S or N159K) were associated with strikingly different mechanisms of action as well as immune and clinical phenotypes. Patients heterozygous for the N159S variant resulting in a DN effect presented with combined immunodeficiency (CID), whereas patients heterozygous for the N159K variant acting through HI presented with CVID. These findings underscore the tight genotype/phenotype correlation between specific mutations, even when impacting the same amino acid, that can result in different T cell, B cell, myeloid, and clinical phenotypes (6, 9).

### IKAROS controls CD45RA and CD45RO expression and regulates HNRNPLL.

**Figure 2:**
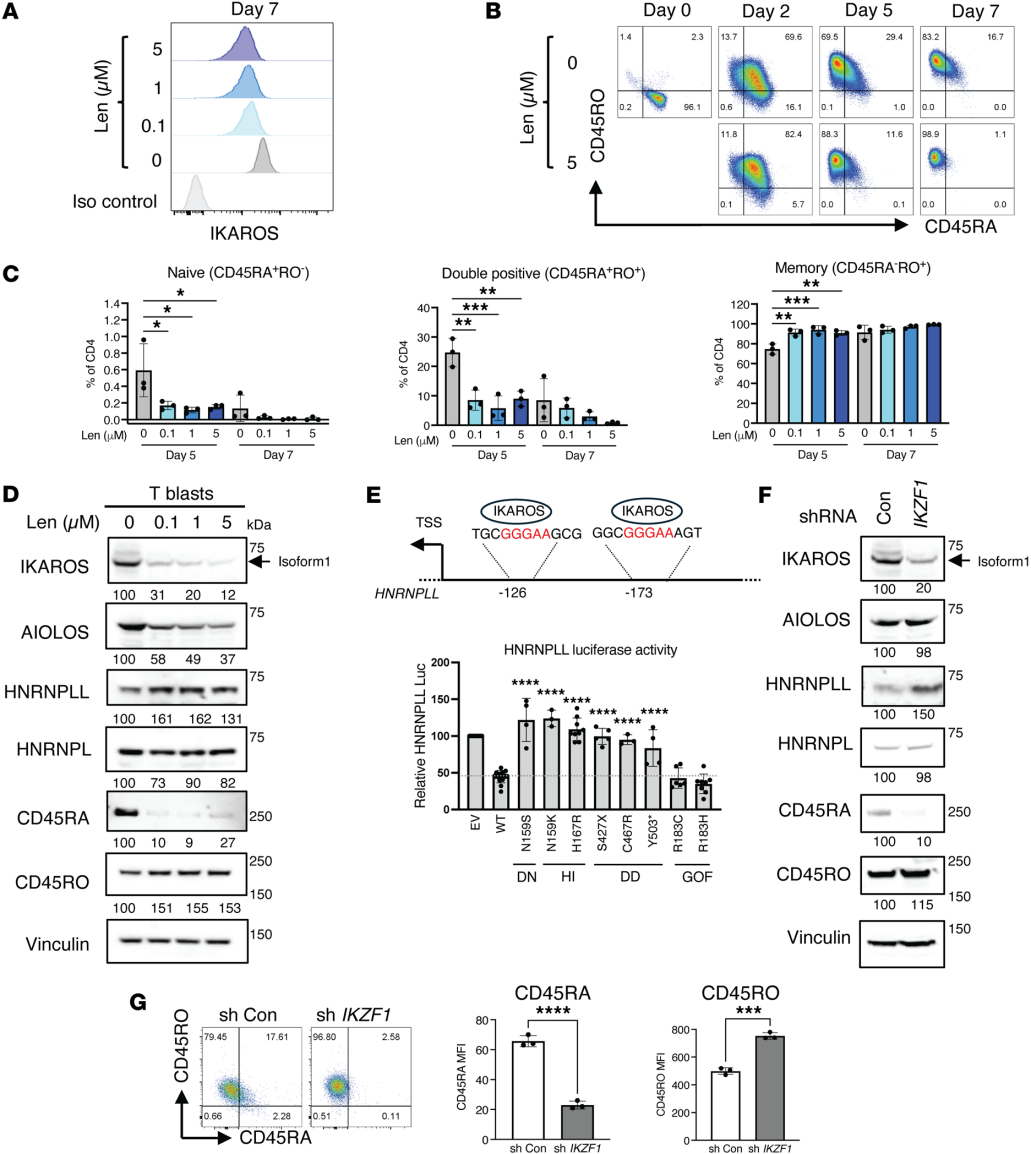
The effect of IKAROS on CD45RA, CD45RO, and HNRNPLL expression. (**A**–**D**) Healthy control naive CD4^+^ T cells were cultured with Dynabeads human T cell activator CD3/CD28 for 7 days in the presence or absence of lenalidomide (0.1, 1, and 5 μM). (**A**) Intracellular IKAROS expression, (**B**) CD45RA and CD45RO expression, (**C**) percentage of naive (CD45RA^+^CD45RO^–^), double-positive (CD45RA^+^CD45RO^+^), and memory (CD45RA^–^CD45RO^+^) cells, and (**D**) immunoblotting data are shown. Data are representative of 3 independent experiments. (**E**) The ReMap ChIP-seq track in the UCSC Genome Browser (https://genome.ucsc.edu; Human ReMap Atlas of Regulatory Regions; fourth release, 2022)] revealed 2 antisense regulatory regions with IKAROS canonical binding sites (GGGAA) upstream of the *HNRNPLL* transcription start site (top). HEK293T cells were cotransfected with HNRNPLL promoter and indicated IKAROS expression plasmids. Empty vector (EV), is represented by the backbone pcDNA3-HA plasmid lacking IKAROS gene sequence information. Gaussia luciferase activity was normalized to secreted alkaline phosphatase. Relative luciferase activity is shown after normalization to EV (bottom). The dotted line indicates the average value for the WT. (**F** and **G**) Healthy control naive CD4^+^ T cells were transduced with lentiviral particles containing GFP control or *IKZF1* shRNA. Three days after infection, GFP^+^ cells were sorted and stimulated with anti-CD3– and CD28–coated beads for 7 days. (**F**) Immunoblot analysis and (**G**) mean fluorescence intensity (MFI) of CD45RA and CD45RO by flow cytometric analysis are shown. For the immunoblot analysis, the numbers below the blots represent densitometry analysis normalized to vinculin. Data represent the mean ± SD from at least 3 independent experiments. Significance was determined using ordinary 1-way ANOVA (Dunnett’s multiple comparisons test), comparing each group to the Len-untreated control (**C**) or WT (**E**), and 2-tailed Student’s *t* test (**G**). *P < 0.05, **P < 0.01, ***P < 0.001, ****P < 0.0001.

We investigated whether IKAROS expression influences the CD45RA-expressing naive and CD45RO-expressing memory T cell phenotype using lenalidomide (Len). In patients with multiple myeloma, and through mechanisms yet to be elucidated, Len has been shown to enhance antigen-specific T cell activation and promote CD45RA downregulation, thereby facilitating the transition from naive to memory/effector phenotypes (14). Len is a thalidomide analog that targets the cereblon (CRBN) E3 ubiquitin ligase complex, modifies its substrate specificity, and promotes IKAROS and AIOLOS proteasomal degradation (15). Treatment with Len at concentrations of 0.1, 1, and 5 μM effectively reduced IKAROS expression in CD4^+^ naive T cells stimulated with CD3/CD28-coated beads, and minimally — but not significantly — affected naive CD4^+^ T cell proliferation (Figure 2A and Supplemental Figure 4A). In HC CD4^+^ naive T cells, Len treatment markedly decreased CD45RA^+^CD45RO^–^- and increased CD45RA^–^CD45RO^+^-expressing cells (Figure 2, B and C). These findings suggest that reduced IKAROS expression promotes a CD45RA^–^/CD45RO^+^ memory-like phenotype, mirroring the pattern observed in patients with HI or DD allelic variants. Western blot analysis further confirmed a reduced CD45RA and increased CD45RO total protein expression on CD4^+^ naive T cells following activation for 7 days in the presence of Len (Figure 2D). Len-mediated IKAROS degradation in activated CD4^+^ T cells was also associated with upregulation of HNRNPLL protein expression; this effect seemed specific, as minimal changes were detected in HNRNPL, an HNRNPLL paralog (Figure 2D). Although less dramatic than CD45RA, CD45 isoforms RB and RC followed the same trend of decreased expression upon Len treatment (Supplemental Figure 4B). Further in silico investigation into the role of IKAROS in regulating *HNRNPLL* transcription by the UCSC Genome Browser revealed the presence of 2 antisense regulatory regions with IKAROS canonical binding sites (GGGAA) upstream of the *HNRNPLL* transcription start site (Figure 2E) (16). When IKAROS-dependent *HNRNPLL* transcriptional activity was biologically tested using a luciferase system, the results confirmed that WT IKAROS negatively regulated *HNRNPLL* transcription. IKAROS LOF variants acting through DN, HI, or DD mechanisms exhibited significantly reduced suppression of *HNRNPLL* transcriptional activity when compared with WT IKAROS; on the other hand, GOF variants showed a trend of enhanced suppression activity when compared with the WT control, although this was not statistically significant (Figure 2E). Interestingly, despite the stark contrast in mechanisms of diseases and T cell phenotypes between N159S (high naive/low memory) and N159K (high memory/low naive), both mutations failed to suppress luciferase activity, suggesting that other factors beyond HNRNPLL may be involved in their T cell immunophenotypes. As previously shown, patients carrying IKAROS DN variants (N159S/T) showed impaired T cell responses, including reduced IL-2–induced STAT5 phosphorylation and anti-CD3–induced T cell proliferation, despite preserved baseline surface expression of IL-2Rα/CD25, IL-2Rβ/CD122, and IL-2Rγ/CD132 (6). When IL-2– and IL-7–induced pSTAT5 phosphorylation was tested in DN N159S and HI N159K patient cells, IL-2 signaling was impaired only in DN N159S cells, while IL-7 signaling was preserved in both allelic variants (Supplemental Figure 4C). Consistent with the findings of the study cited above (6), TCR-induced T cell proliferation was impaired in cells from the N159S patient, but not in cells from the N159K patient (Supplemental Figure 4C). These results indicate that the cytokine signaling defect was selective/allelic variant dependent, and the impaired function of the IL-2 pathway is likely linked to the T cell activation, expansion, and differentiation defects observed in DN N159S cells. These results also suggest that, not only IKAROS dysfunction and HNRNPLL, but also the integrity of T cell cytokine response likely affects T cell activation, leading to defective naive/memory cell formation and Th differentiation.

As Len promotes both IKAROS and AIOLOS degradation, we selectively silenced IKAROS using small hairpin RNA (shRNA) to assess its independent effect on HNRNPLL-regulated CD45RA and CD45RO expression. HC CD4^+^ naive T cells were transduced with lentiviral particles containing *IKZF1* shRNA, and efficiently transduced cells were sorted and cultured with IL-2 and anti-CD3/CD28–coated beads for 7 days before final evaluation. Western blot analysis confirmed that IKAROS silencing (~80%) was accompanied by an increase on HNRNPLL and CD45RO expression, whereas CD45RA levels decreased. No remarkable effects were observed on AIOLOS or HNRNPL expression, underscoring the specificity of IKAROS silencing and its downstream effects (Figure 2F). The effects of IKAROS silencing in HC CD4^+^ naive peripheral T cells were confirmed by flow cytometry, which showed reduced surface CD45RA median fluorescence intensity (MFI) and increased CD45RO MFI (Figure 2G).

These results show that the two in vitro models of reduced IKAROS expression, i.e., by Len-induced degradation and transcriptional inhibition by shRNA, resulted in increased HNRNPLL expression, along with reduced CD45RA and increased CD45RO cell surface and total protein expression. It is noteworthy that while Len induced IKAROS and AIOLOS degradation, the shRNA experiment specifically and exclusively targeted IKAROS. As the shRNA results did not differ from those of the Len experiments, this indicates that IKAROS has a direct and leading role in controlling HNRNPLL and, in turn, CD45RA/RO expression and naive/memory peripheral T cell phenotypes.

In a sequential model, these findings suggest that IKAROS binds to the *HNRNPLL* promoter and regulates its transcription and protein levels. Then, HNRNPLL, by itself or more likely in combination with other intrinsic cellular factors, determines different naive/memory T cell ratios in patients with IKAROS-associated diseases. This effect, as described in the section above and in Figure 1 and Supplemental Figure 2, was more evident in CD4^+^ T cells than in CD8^+^ T cells, also suggesting different lineage sensitivities to the IKAROS-HNRNPLL axis and CD45RA/RO regulation.

### IKAROS contributes to T cell cytokine production and Th commitment regulation.

Patients with IKAROS-associated diseases predominantly present with distinctive T cell cytokine production patterns and Th phenotypes. IKAROS DN mutation carriers have a strongly skewed Th0 phenotype (T cells with limited IL-2 but virtually absent IL-4 or IFN-γ production); and IKAROS GOF mutation carriers present with a Th2 high/Th1 low–skewed phenotype. IKAROS patients with HI or DD defects have less biased cytokine production and Th commitment phenotypes (3, 6, 10).

**Figure 3:**
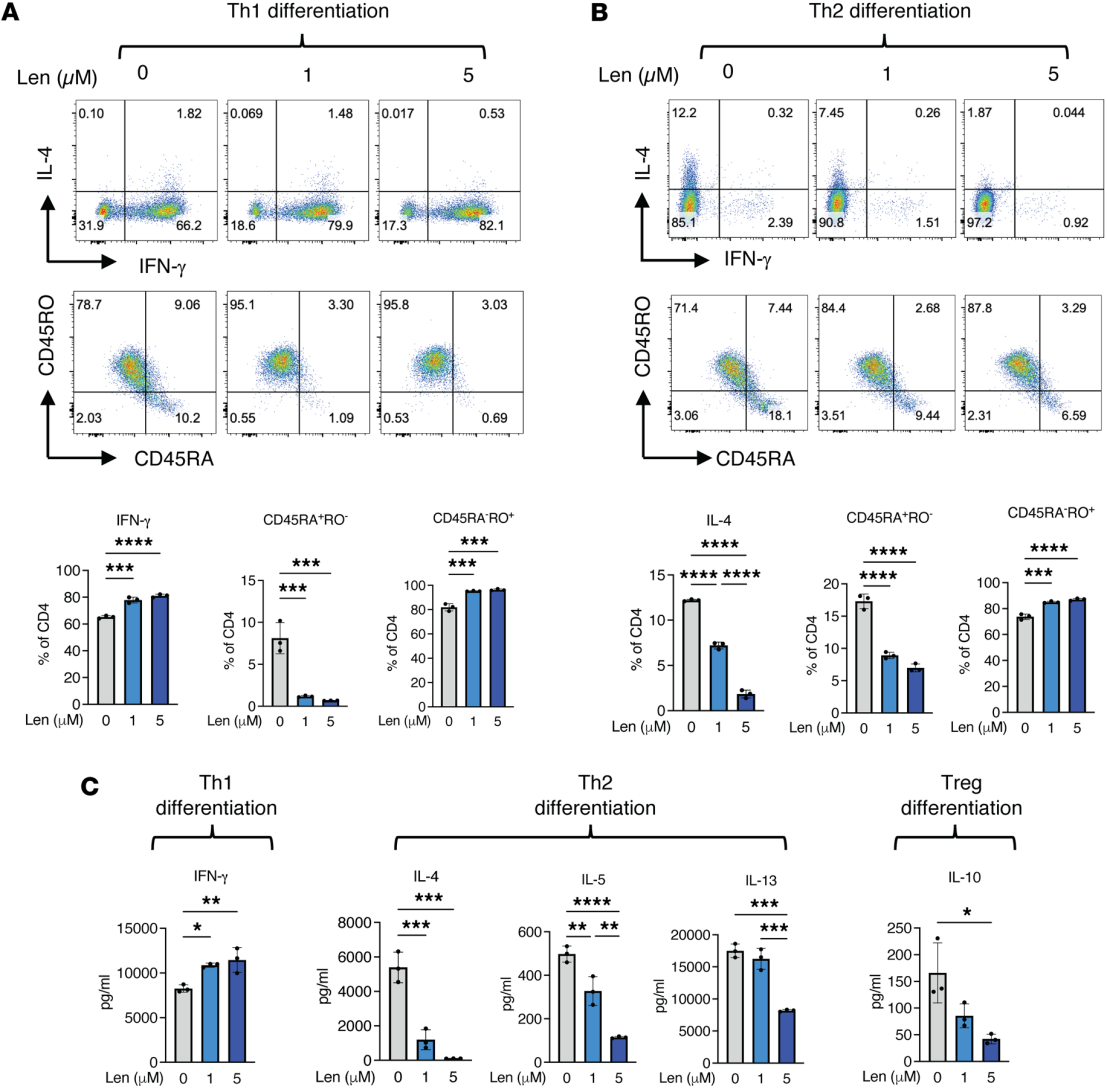
Degradation of IKAROS enhances Th1 differentiation but suppresses Th2 differentiation. (**A** and **B**) Naive CD4^+^ T cells from healthy controls were cultured with plate-bound anti-CD3 and soluble anti-CD28 for 8 days along with differentiating cytokines (IL-12, IL-2, and anti–IL-4 antibody for Th1 cells and IL-4, IL-2, and anti–IFN-γ antibody for Th2 cells) in the presence or absence of lenalidomide (1–5 μM). Cells were rested for 4 days without anti-CD3/CD28 stimulation but with differentiating cytokines and lenalidomide. Cells were then stimulated with PMA, ionomycin, and brefeldin A for 5–6 hours, stained with CD45RA and CD45RO and intracellular IFN-γ and IL-4, and analyzed by flow cytometry. (**C**) Cells were stimulated as in **A** and **B** but without brefeldin A for 24 hours. TGF-β, IL-2, anti–IFN-γ, and anti–IL-4 were used for Treg differentiation. Measurement of secreted cytokines by Luminex from the supernatants of Th1 cells (IFN-γ), Th2 cells (IL-4, IL-5, and IL-13), and Tregs (IL-10). Data represent the mean ± SD from at least 3 independent experiments. Significance was determined using ordinary 1-way ANOVA (Tukey’s multiple comparisons test). *P < 0.05, **P < 0.01, ***P < 0.001, ****P < 0.0001.

To explore the effect of IKAROS on cytokine production and Th commitment, purified HC CD4^+^ naive peripheral T cells were cultured in Th1- or Th2-specific differentiation conditions for 12 days in the absence or presence of Len, an IKAROS immunomodulator. Following phorbol 12-myriate 13-acetate (PMA) and ionomycin stimulation, IFN-γ and IL-4 were measured by flow cytometric intracellular staining; in addition, IFN-γ, IL-4, IL-5, IL-13, and IL-10 were quantified in culture supernatants by Luminex. As shown in Figure 2B, Len-mediated IKAROS degradation resulted in a reduced CD45RA and augmented CD45RO expression in Th-uncommitted environments. Remarkably, opposite trends were detected for Th1- and Th2-specific cytokines, where Th1-associated IFN-γ was increased by approximately 20% and Th2-associated IL-4 was decreased by approximately 85% in a Len-mediated dose-dependent manner (Figure 3, A and B). Cytokine concentrations in the supernatants further confirmed the increased Th1 (IFN-γ) and decreased Th2 (IL-4, IL-5 and IL-13) cytokine secretion, as well as decreased Treg-associated IL-10, in a Len-mediated dose-dependent manner (Figure 3C).

These results suggest that IKAROS influences T cell cytokine production and secretion, as well as Th cell commitment, with a more pronounced effect over Th2 cells than Th1 cells. We further examined T-bet and GATA3, the key transcription factors for Th1 and Th2 differentiation, under both Th1 and Th2 differentiation conditions. Naive CD4^+^ T cells from individuals acting as healthy controls under Th1 differentiation conditions did not show any relevant Len effect on T-bet or GATA3 expression; on the other hand, naive CD4 T^+^ cells under Th2 differentiation conditions showed an increased trend on T-bet expression but no effect on GATA3 (Supplemental Figure 5). Previous studies have shown that IKAROS directly occupies the *tbx21* promoter, with strong binding under Th2-polarizing conditions but not under Th1-polarizing conditions. Loss of IKAROS activity disrupts this repression and allows T-bet expression during Th2 differentiation (17). While our data are consistent with these findings, increased T-bet expression by itself is unlikely to explain the reduction in Th2 differentiation; further studies will be needed to identify other contributing mechanisms. Moreover, IKAROS is unlikely to be the sole regulator of T cell cytokine production, secretion, and Th cell commitment, as while IKAROS GOF patients exhibit a Th2-biased immunophenotype, IKAROS modulation by Len did not fully recapitulate the Th cytokine profile observed in IKAROS DN patients (3, 6, 10).

### IKZF1 DN and HI variants are associated with abnormal T cell development in the ATO system.

**Figure 4:**
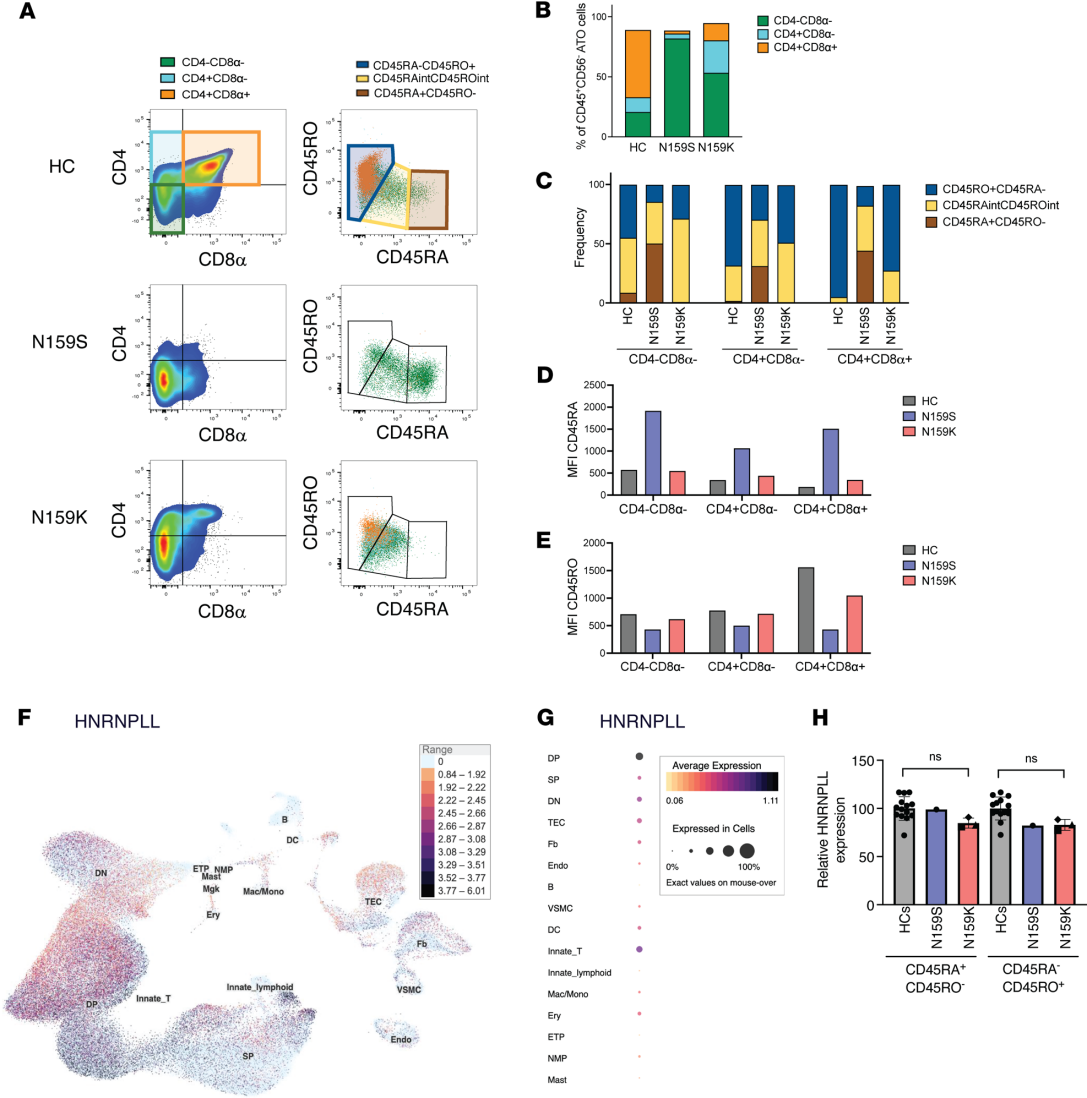
Mutations in IKZF1 lead to altered T cell development in the thymus. (**A**) Representative flow cytometry analysis of T cell differentiation in a healthy donor (HC), a IKZF1 DN patient (N159S), and a IKZF1 HI patient (N159K) after 6 weeks of culture in the ATO system (*n* = 1). Cells were gated on live/dead^–^CD45^+^CD56^–^ cells and stained for CD4, CD8a, CD45RA, and CD45RO. The left column shows the progression of T cell differentiation from CD4^–^CD8^–^ double-negative (CD4^–^CD8^–^, green box), to immature CD4^+^ (CD4^+^CD8^–^, turquoise box) to CD4^+^CD8^+^ double-positive (CD4^+^CD8^+^, orange box) cells in the ATO system. The right column shows CD45RA and CD45RO expression in the same 3 subsets. (**B**) Bar graph showing the frequency of CD4^–^CD8^–^, CD4^+^CD8^–^, and CD4^+^CD8^+^ cells in ATOs of the HC and patients. (**C**) Bar graph showing the frequency of CD45RA^+^CD45RO^–^ (brown gate), CD45RA^int^CD45RO^int^ (yellow gate), and CD45RA^–^CD45RO^+^ (blue gate) cells among CD4^–^CD8^–^, CD4^+^CD8^–^, and CD4^+^CD8^+^ cells in ATOs of the HC and patients. (**D** and **E**) Bar graphs showing the mean fluorescence intensity (MFI) of CD45RA (**D**) and CD45RO (**E**) expression in CD4^–^CD8^–^, CD4^+^CD8^–^, and CD4^+^CD8^+^ cells from the ATOs of the HC and patients. (**F** and **G**) UMAP (**F**) and dot plot (**G**) showing the expression level of *HNRNPLL* in the different cell subsets of the human thymus atlas. The data was obtained from the collection of single-cell datasets available on the UCSC Cell Browser (https://fetal-thymus.cells.ucsc.edu) (33) (**H**) HNRNPLL MFI values in CD4 cells were normalized to the average of corresponding HC subset (set to 100%), and relative protein expression is displayed. Data are from 3 experiments; N159K patient points reflect the average of 2–3 replicates. Mutation N159S was studied in patient A.I.1; mutation N159K was studied in patients B.I.1 (diamond), B.II.1 (square) and B.II.2 (triangle). Gray boxes represent HC values from 12 individuals. Significance was determined using ordinary 1-way ANOVA (Dunnett’s multiple comparisons test).

To further characterize the role of IKAROS in early T cell development, we differentiated CD34^+^ hematopoietic and stem progenitor cells into T cells in the ATO system from 2 patients carrying different missense IKAROS allelic variants affecting the same codon (N159S and N159K) but exerting different effects (DN and HI, respectively). To this purpose, CD34^+^ cells were isolated from PBMCs of patients and individuals acting as healthy controls, aggregated with MS5-hDLL4 stromal cells, followed by culture in RB27-, IL-7–, and FLT3L-containing medium for 5 weeks before evaluation. Progression of T cell development was assessed by flow cytometry, by staining for CD4 and CD8 expression (Figure 4A), to define CD4^–^CD8^–^ double-negative, immature CD4^+^ (CD4^+^CD8^–^), and CD4^+^CD8^+^ double-positive T cells. CD45RA and CD45RO expression was analyzed at each developmental stage in patients and individuals acting as healthy controls (Figure 4A). Previous studies have demonstrated that progression of T cell development in the thymus from double-negative to double-positive cells is marked by transitioning from CD45RA to CD45RO expression (18, 19). HC CD34^+^ cells were able to differentiate in the ATO system from CD4^–^CD8^–^ double-negative to immature CD4^+^ and finally to CD4^+^CD8^+^ double-positive T cells (Figure 4, A and B). This differentiation was also accompanied by a progressive shift from CD45RA to CD45RO expression (Figure 4, A–E). By contrast, cells carrying the DN N159S mutant showed a predominant block at the CD4^–^CD8^–^ double-negative cell stage, and the vast majority of them retained high levels of CD45RA expression (Figure 4, A–E). In comparison, the HI N159K mutant allowed differentiation to immature CD4^+^ cells and, to a minor extent, to the CD4^+^ CD8^+^ double-positive stage. Consistent with this, the N159K developing T cells showed intermediate levels of CD45RA and CD45RO expression (Figure 4, A–E). The observation that developing T cells carrying the N159S mutant are predominantly CD45RA^+^ recapitulated the peripheral blood T cell phenotype for this allelic variant but contrasted with the expectation that this DN IKAROS mutant should unleash HNRNPLL activity, ultimately promoting CD45RO expression. To investigate a possible reason for this discrepancy, we interrogated a publicly available resource of gene transcription in the human thymus and observed that *HNRNPLL* was more abundantly expressed starting at double-positive stage of T cell development (Figure 4, F and G) (20). These data strongly suggest that IKAROS regulates early stages of T cell development through mechanism(s) independent of HNRNPLL, and therefore, the peripheral blood T cell phenotypes in IKAROS-associated diseases can have thymic as well as postthymic contributions. In fact, while HNRNPLL expression was reduced in N159K CD45RA^+^/RO^–^, as well as in N159K and N159S CD45RA^–^/RO^+^ patient cells, none of those differences were statistically significant when compared with the healthy controls (Figure 4H), also pointing at HNRNPLL-independent mechanisms of CD45RA/CD45RO expression regulation.

Based on our results, we provide evidence in support of IKAROS-dependent effects on T cells both during early development (through the ATO studies) and in the periphery (as shown by Len, shRNA, and HNRNPLL studies). It is noteworthy that these IKAROS-dependent effects on T cells are IKAROS allelic variant dependent and a contributory part of a broader and previously established set of factors regulating T cell development, phenotype, and function (3, 6, 10).

## Discussion

Herein, we present evidence that IKAROS regulates T cell naive/memory immunophenotype and function in a mechanism-of-disease-specific way — with a more pronounced effect in CD4^+^ T cells than in CD8^+^ T cells — as well as HNRNPLL expression.

IKAROS involvement on T cell naive/memory immunophenotype was determined by different lines of evidence. We first conducted a retrospective/prospective evaluation of peripheral blood T cell immunophenotypes in patients with IKAROS-associated diseases due to HI, DD, DN, or GOF mechanisms of disease. Through this approach, distinctive T cell naive/memory distribution patterns were detected. DN and GOF patients primarily displayed increased CD45RA^+^ naive and TEMRA cells/decreased CD45RA^–^ CM cells, while patients with HI and DD allelic variants presented the opposite profile with increased CD45RA^–^ CM cells/decreased CD45RA^+^ naive and TEMRA cells. Of note, while all patients carrying IKAROS GOF mutations presented with similar clinical manifestations, their immunophenotypes were more variable. Patients carrying R183C and T398M variants showed a trend toward high naive/low EM CD4^+^ T cells, while R183H patients exhibited a more dispersed pattern. Different factors, genetic (e.g., subtle different mechanisms of action involved, even when mutations affect the same amino acid), environmental (e.g., all R183C patients belong to the same family), interventional (e.g., most IKAROS GOF patients are under strong and different immunomodulatory therapies), or other variables could have influenced these results. Len-mediated biochemical reduction of IKAROS and IKAROS gene silencing by shRNA, both interventions oriented to mimic LOF variants, resulted in a dramatic decrease in CD45RA expression. While these results correlate with the peripheral blood findings in patients carrying LOF variants acting by HI and DD, they do not explain the pattern observed in the DN patients, who have another form of LOF mutation. It is noteworthy that in the same Len and shRNA studies, we also detected a specific decrease in HNRNPLL expression (i.e., HNRNPLL’s paralog HNRNPL was not impacted), along with increased CD45RO accumulation, suggesting that IKAROS regulates HNRNPLL expression and CD45RA-to-CD45RO transition.

The role of HNRNPLL as the master regulator of CD45RA naive/CD45RO memory expression maturation has been well established since 2008 (8). HNRNPLL is an RNA-binding tyrosine phosphatase preferentially expressed in activated T lymphocytes and plasma cells, while its paralog HNRNPL is ubiquitously expressed. In resting human naive T cells, HNRNPLL expression is limited but is upregulated upon T cell receptor stimulation. The increased HNRNPLL expression is followed by CD45 splicing, switching from higher-to-lower molecular-weight isoforms. With elevated HNRNPLL expression, T cells lose CD45RA expression (the largest CD45 isoform and naive cell marker) and gain the memory cell marker CD45RO (a CD45 isoform resulting from loss of all 3 alternative exons) (8, 21). Through our investigations, we were able to further and directly connect IKAROS and HNRNPLL. Upon reviewing *HNRNPLL* promoter region, in silico analysis identified 2 IKAROS canonical binding sites (GGGAA); moreover, the publicly accessible ChIP-Atlas database shows IKAROS binding at the *HNRNPLL* promoter in multiple datasets (https://chip-atlas.org/peak_browser). In addition, when biologically tested in a luciferase system, WT IKAROS was found to act through the *HNRNPLL* promoter region and functioned as a transcriptional suppressor. Correspondingly, when compared with WT IKAROS, LOF variants acting through DN, HI, and DD mechanisms exhibited significantly reduced suppression of the HNRNPLL transcriptional activity, whereas GOF variants showed a trend of enhanced suppression activity, although the latter was not statistically significant. Thus, even though our experiments show that IKAROS regulates HNRNPLL and controls CD45RA/CD45RO expression on T cells, a linear model where IKAROS regulates HNRNPLL, which, in turn, regulates CD45RA-to-CD45RO transition does not seem to fully or unequivocally explain the mechanisms described in this study.

Len has been shown to promote the degradation of both IKAROS and AIOLOS, and in its turn to influence Th cell commitment, promoting Th1 polarization (15, 22, 23). Quintana et al. reported that T cells from Aiolos-deficient mice produce more IL-2 and exhibit impaired Th17 differentiation under Th17-polarizing conditions, while under Th1-polarizing conditions they display increased expression of Tbx21 (T-bet) and IFN-γ, demonstrating that Aiolos contributes to Th17 differentiation while limiting Th1 generation in murine models (24). When focused on patients with AIOLOS-associated diseases, they display variable and nonconsistent T cell immunophenotypes. Patients with AIOLOS E82K and Q402* mutations acting by AIOLOS HI had naive/memory T cell subsets and Th1/2/17 frequencies comparable to those of individuals acting as healthy controls (25). In contrast, patients carrying AIOLOS G159R and N160S mutations, both heterodimerizing with IKAROS and acting by AIOLOS DN mechanisms, presented with distinct immune and clinical presentations and largely opposite T cell phenotypes: G159R was associated with low CD45RA/high CD45RO and increased Th1 with reduced Th17, whereas N160S showed high CD45RA/low CD45RO, with globally reduced Th1/Th2/Th17 frequencies (26, 27). Altogether, although AIOLOS is degraded by Len and this may influence Th differentiation, its direct mechanism of action and fundamental role in regulating human T cell immunophenotypes remains to be defined.

Our ex vivo studies exploring the effects of IKAROS on early stages of T cell development in the ATO system further support the concept of a less than linear and more complex/multilayer mechanism involved in IKAROS regulation of CD45RA-to-CD45RO transition. The two IKAROS allelic variants evaluated, DN and HI — both LOF although acting by different mechanisms — recapitulated at different levels the patients’ peripheral blood T cell immunophenotypes. The early T cell development of the IKAROS-DN precursor cells showed a block at CD4^–^CD8^–^ double-negative stage and a CD45RA^+^-biased profile, highly consistent with the peripheral blood results; on the other hand, the IKAROS-HI precursor cell development showed a more mature but still abnormal phenotype, enriched at CD4^–^CD8^–^ double-negative and immature CD4^+^ stages, with cells coexpressing intermediate levels of CD45RA and CD45RO (CD45RA^int^CD45RO^int^), partially mimicking the peripheral blood profile. More importantly, the 2 different IKAROS-dependent early T cell development phenotypes observed in the ATO are most likely HNRNPLL independent as the master regulator of CD45RA-to-CD45RO transition is more abundantly expressed at the double-positive cell stage, downstream from the CD4^–^CD8^–^ double-negative enriched patterns detected. In other words, a more impactful role of IKAROS on HNRNPLL expression and T cell differentiation, function, and phenotype can be expected only at late stages of intrathymic differentiation and in the periphery.

It is noteworthy that other critical IKAROS-dependent/HNRNPLL-independent T cell intrinsic dysfunctional features distinguish IKAROS-DN and IKAROS-HI and can affect both thymic and peripheral T cell development, phenotype, and function. In fact, patients carrying IKAROS DN variants showed impaired T cell responses, including reduced IL-2–induced STAT5 phosphorylation and soluble anti-CD3/CD28–induced T cell proliferation (6). When we evaluated T cell proliferation, IL-2 signaling (IL-2Rα/CD25, IL-2Rβ/CD122, and IL2Rγ/CD132 dependent) and IL-7 signaling (IL-7Ra/CD127 and IL2Rg/CD132 dependent) in patient cells carrying either N159S or N159K mutations, we found that N159S cells showed impaired TCR-induced T cell proliferation and IL-2 signaling, with preserved IL-7 signaling, whereas under the same testing conditions all N159K cell responses were comparable to healthy controls (Supplemental Figure 4C). These results suggest that, not only IKAROS dysfunction and HNRNPLL regulation, but also the integrity of T cell cytokine response likely affects T cell activation, Th differentiation, and naive/memory cell formation (28–30).

The differences observed between IKAROS mutations N159S and N159K go far beyond two missense changes affecting the same amino acid and deserve a separate analysis. These changes not only resulted in discrepant mechanisms of diseases, but also clinical phenotypes — e.g., DN/CID versus HI/CVID — by impacting a single *IKZF1* mutational hotspot (6, 9). This observation highlights the need for independent in vitro functional validation of different missense changes, even when affecting the same codon.

In summary, our research demonstrates how IKAROS impacts human T cell immunophenotype and function. This conclusion is supported by multiple lines of evidence, including studies of patients with IKAROS-associated diseases carrying different allelic variants that act through HI, DD, DN, and GOF mechanisms; functional modulation of the IKAROS protein via Len treatment or shRNA-mediated gene silencing; and studying early T cell development using an ATO system. Altogether, our findings provide a comprehensive understanding of the role of IKAROS in T cell development and differentiation. T cell phenotyping in patients carrying IKAROS variants of uncertain significance, along with the assessment of their impact on B cells, will provide valuable insights for the functional interpretation of these genetic alterations and their relevance to immune dysregulation.

## Methods

### Sex as a biological variable.

Our study examined male and female individuals, and similar findings are reported for both sexes.

### Cell culture and expansion of T cell blasts.

Ficoll (Cytiva) density gradient centrifugation was used to isolate PBMCs. Cells were cultured in RPMI 1640 supplemented with 10% FBS and penicillin/streptomycin (complete medium). HEK-293T (ATCC, CRL-3216) and NIH-3T3 cells (ATCC, CRL-1658) were cultured in Dulbecco’s modified Eagle’s medium supplemented with 10% FBS and penicillin/streptomycin. Naive CD4^+^ T cells were enriched using a negative isolation kit (Stem Cell Technologies, catalog 19555) and cultured in complete RPMI 1640 medium with Dynabeads Human T-Activator CD3/CD28 (Thermo Fisher, catalog 11131D) in the presence or absence of Len (0.1–5 mM) (Sigma-Aldrich, catalog 901558) for 7 days.

### Flow cytometry.

For lymphocyte phenotyping, either whole blood or PBMCs were incubated with the following mAbs: anti-CD3 (clone S4.1[7D6], Life Technologies), anti-CD4 (clone RPA-T4, BD Biosciences), anti-CD8 (clone RPA-T8, BD Biosciences), anti-CD45RA (clone ALB11, Beckman Coulter or clone HI100, BD Biosciences), anti-CD45RO (clone UCHL1, Dako), CD45RB (clone MEM-55, Biolegend), CD45RC (clone MT2, BD Biosciences), and anti-CD62L (clone DREG-56, BD Biosciences) for 30 minutes at 4°C. Red blood cells were lysed using FACS Lysing Solution (BD Biosciences, catalog 349202). For intracellular staining, cells were stained with LIVE/DEAD stain (Thermo Fisher, catalog L34976). Then, cells were fixed and permeabilized with a FOXP3 staining kit (Thermo Fisher, catalog 00-5523-00) and were stained with the following mAbs: anti–IFN-γ (clone B27, Biolegend), anti–IL-4 (clone MP4-25D, Biolegend), anti–IL-10 (clone JES3-9D7, Biolegend), anti-IKAROS (clone R32-1149, BD Biosciences), anti-HNRNPLL (ABC Biolab, catalog ABCCS48618), anti-T-bet (clone O4-46, BD Biosciences, catalog 561316), and anti-GATA3 antibodies (clone TWAJ, Invitrogen, catalog 53-9966-42). Cells were analyzed with BD FACS Canto II or FACS Lyric (BD) flow cytometer, and data were processed with FlowJo software (Tree Star Inc., version 10.8.1).

### Th subset differentiation and cytokine measurements.

Naive CD4^+^ T cells (CD4^+^CD45RA^+^CD45RO^–^) were isolated from PBMCs using a negative isolation kit (Stem Cell Technologies, catalog 19555). The purity of naive T cells was >90%. Naive CD4^+^ T cells were cultured in anti-CD3–coated plates (5 μg/mL) (Thermo Fisher, catalog 16-0037-85) plus soluble anti-CD28 mAb (2 μg/mL) (Thermo Fisher, catalog 16-0289-85), IL-2 (100 IU/mL) (Peprotech, catalog 200-02), and differentiating cytokines in the presence or absence of Len (1 or 5 μM) (Sigma-Aldrich, catalog 901558); IL-12 (10 ng/mL) (Peprotech, catalog 200-12H) and anti–IL-4 (10 μg/mL) (BD Biosciences, catalog 554481) for Th1; IL-4 (10 ng/mL) (Peprotech, catalog 200-04) and anti–IFN-γ (10 μg/mL) (BD Biosciences, catalog 554547) for Th2; and TGF-β (10 ng/mL) (Peprotech, catalog 100-21), anti–IFN-γ, and anti–IL-4 for Tregs. After the 8 days of culture, cells were transferred to plates without anti-CD3 and anti-CD28 stimulation and maintained with IL-2 plus the specific Th differentiation cytokines for 4 more days in the presence or absence of Len.

For cell-based cytokine measurement, the T cell blasts were restimulated with PMA (100 ng/mL) (Sigma-Aldrich) and ionomycin (1 μg/mL) (Sigma-Aldrich) for 5–6 hours at 37°C in the presence of brefeldin A (5 μg/mL) (Sigma-Aldrich). After surface staining for CD45RA and CD45RO, cells were fixed and permeabilized with the BD Cytofix/Cytoperm Plus Kit (BD Pharmingen) and then stained with anti–IFN-γ (B27, Biolegend) and anti–IL-4 (MP4-25D, Biolegend). Cells were analyzed with BD FACS Canto II flow cytometer, and data were processed with FlowJo software.

For secreted cytokine measurements, the T cell blasts were restimulated with PMA (50 ng/mL) and ionomycin (1 μg/mL) for 24 hours. Culture supernatants were collected and stored at –20°C. Cytokines in the supernatants were measured using the Human ProcartaPlex kit (Thermo Fisher, catalog EPX180-12165-901) according to the manufacturer’s instructions. The samples were acquired in a Bio-plex 200 system, and cytokine concentrations were calculated using the Bio-Plex manager software (Luminex xPonent) with a 5-parameter curve-fitting algorithm applied for standard curve calculation.

### Proliferation assay.

To test the patient’s T cell proliferation, total PBMCs were incubated with CellTrace Violet dye (1 μM) (Thermo Fisher, catalog C34557) for 20 minutes at 37°C in a humidified 5% CO_2_ incubator. Celltrace Violet–stained cells were washed 2 times with complete RPMI medium and stimulated with soluble anti-CD3 and anti-CD28 (1 μg/mL each, catalog 16-0037-85 and 16-0289-85, eBioscience). After 4 days of incubation, cells were stained with fluorochrome-conjugated CD4 (clone RPA-T4, BD Biosciences) and CD8 antibodies (clone RPA-T8, BD Biosciences) and acquired by flow cytometry (BD FACS Canto II). To test the effect of Len on T cell proliferation, enriched naive CD4 T cells were stimulated with Dynabeads Human T-Activator CD3/CD28 beads in the presence or absence of Len (0.1, 1, or 5 mM) for 4 days.

### Measurement of phospho-STAT5.

PBMCs were either left unstimulated or stimulated with IL-2 (10 ng/mL, Peprotech, catalog 200-02) or IL-7 (10 ng/mL, Peprotech, catalog 200-07) for 20 minutes in the presence of APC anti-CD4 antibody (BD Biosciences, catalog 555349) at 37°C. Cells were fixed using BD Cytofix Fixation Buffer (BD Biosciences, catalog 554655) for 15 minutes at 37°C and then permeabilized in BD Phosflow Perm Buffer III (BD Biosciences, catalog 558050) for 30 minutes on ice. Cells were washed twice with stain buffer (BD Biosciences, catalog 554656) and then stained with phospho-STAT5 (Y694) antibody (BD Biosciences, catalog 560311) for 1 hour. After staining, cells were washed twice with stain buffer and analyzed on a BD FACSCanto II flow cytometer. For STAT5 phosphorylation analysis, CD4 T cells were gated.

### Lentiviral transduction and IKAROS knockdown.

Naive CD4^+^ T cells were spin infected with lentivirus containing *IKZF1* shRNA or a control at an MOI of 25 (Origene, catalog TL308127V). Briefly, 1 × 10^6^ naive CD4^+^ T cells from individuals acting as healthy controls were activated with Dynabeads Human T-Activator CD3/CD28 beads (Thermo Fisher, catalog 11131D). After 24 hours, concentrated virus was added, and the cells were spin infected at 1,000*g* for 90 minutes in the presence of polybrene (8 μg/mL) (MilliporeSigma, catalog TR-1003). Following infection, the virus was removed and fresh complete media along with 100 IU of IL-2 (Peprotech, catalog 200-02) was added. Three days after infection, GFP^+^ cells were sorted to 90% purity and further cultured in complete media for 7 days. Surface staining was performed with CD45RA (BD Biosciences, catalog 555488) and CD45RO (BD Biosciences, catalog 555493), and flow cytometry was performed on a BD FACS Canto II.

### Immunoblotting.

Total protein lysates were prepared using lysis buffer (10 mM Tris [pH 7.8], 150 mM NaCl, 1 mM EDTA, 1% NP-40, and protease inhibitor cocktail [Sigma, PPC1010-5ml]) or RIPA buffer. Proteins were separated by NuPAGE Novex 4%–12% Bis-Tris Protein Gels (Life Technology) and transferred to nitrocellulose membranes using a Trans-Blot Turbo Transfer system (Bio-Rad). The membranes were incubated with anti-IKAROS (Cell signaling, catalog 14859), anti-AIOLOS (Cell signaling, catalog 15103), anti–HNRNPLL (Cell signaling, catalog 4783), anti–HNRNPL (Cell signaling, catalog 37562), anti-CD45RA (Santa Cruz, catalog sc-19664), anti-CD45RO (Santa Cruz, catalog sc-1183), or anti-vinculin (Santa Cruz, sc-73614), followed by horseradish peroxidase–conjugated secondary antibodies, and the target proteins were developed by use of SuperSignal West Dura Extended Duration Substrate (Thermo Fisher Scientific, 34076). The images were acquired and analyzed with iBright Imaging system (Thermo Fisher Scientific).

### Luciferase assay.

HEK293T cells were transfected with indicated pcDNA3-HA-IKAROS WT, pcDNA3-HA-IKAROS mutants, or pcDNA3-HA lacking IKAROS gene sequence (referred as empty vector), together with the HNRNPLL promoter plasmid (Genecopoeia, catalog HPRM58581-PG04) using a Effectene transfection kit according to the manufacturer’s instructions (Qiagen, catalog 301427). After 20–24 hours, cell supernatants were collected and analyzed for luciferase activity (Genecopoeia, catalog LF032) according to the manufacturer’s protocol.

### Generation of ATOs.

The ATOs were generated by aggregating a DLL4-expressing stromal cell line (MS5-hDLL4) with CD34^+^ cells isolated from fresh peripheral blood of a healthy individual and cryopreserved peripheral blood of patients carrying IKZF1 mutations, using the CD34 Microbead kit Ultrapure (Miltenyi Biotech) on the Auto MACS Pro Separator. The ATOs were generated and cultured as previously described with few adjustments (31, 32). Briefly, 800–1,000 CD34^+^ cells were combined with 150,000 MS5-hDLL4 cells per ATO. Each ATO (5 μL) was then plated in a 0.4 μM Millicell Transwell insert, placed on a well of a 6-well plate containing 1 mL complete RB27 medium supplemented with rhIL-7 (5 ng/mL), rhFlt3-L (5 ng/mL), and 30 μM l-ascorbic acid 2-phosphate magnesium salt hydrate. Each insert contained a maximum of 2 ATOs. Importantly, for the first 3 weeks of culture, the medium was also supplemented with 10 ng/mL of rhSCF. After 5–6 weeks in culture, ATOs were collected by adding MACS buffer (PBS with 0.5% BSA and 2 mM EDTA) to each well and pipetting to dissociate the ATOs. Cells were then pelleted, resuspended in FACS Buffer (PBS 2% FBS), counted, and stained with the following antibodies: TCRab PE (clone IP26, Biolegend), CD4 APC-Cy7 (clone SK3, BD Biosciences), CD56 PE/Dazzle 594 (clone HCD56, Biolegend), CD45 V500 (clone HI30, BD Biosciences), CD3 BV421 (clone UCHT1, BD Biosciences), CD8a PE-Cy7 (clone RPA-T8, BD Biosciences), CD45RA FITC (clone HI100, Biolegend), CD45RO PerCP-Cy5.5 (clone UCHL1, Biolegend), and LIVE/DEAD Fixable Yellow Dead Cell Stain Kit (Invitrogen). Events were acquired on a BD LSR II Fortessa (BD Biosciences) and analyzed using FlowJo software (FlowJo LLC).

### Electrophoresis mobility shift assay.

HEK293T cells were transfected with Flag expression vectors for IKAROS WT and/or the indicated mutants in 60 mm dishes using Effectene (Qiagen, catalog 301427). After 48 hours of incubation, nuclear extracts were prepared using the NE-PER nuclear and cytoplasmic extraction kit (Thermo Fisher Scientific, catalog 78835). Nuclear extracts (1–2 μg) were subjected to gel mobility shift assays by using LightShift Chemiluminescent EMSA kit (Thermo Fisher Scientific, catalog 20148) according to the manufacturer’s instructions. 6% Novex TBE gels were used for the assay. A biotinylated gSat8 probe was used for the EMSA assay (forward 5′-BIOTIN, GCGAGACCGCAGGGAATGCTGGGAGCCTCCC; reverse 5′-BIOTIN, GGGAGGCTCCCAGCATTCCCTGCGGTCTCGC). The images were acquired using the IBright FL1500 Imaging System and analyzed with iBright Analysis Software (Thermo Fisher scientific).

### Pericentromeric heterochromatin localization.

NIH3T3 cells were transfected with HA-tagged IKAROS WT or the mutants with or without Flag tagged IKAROS WT using a Lonza Nucleofector kit R (catalog VCA-1001, program A-24) and cultured on cover slips in 6-well plates. The next day, cells were washed twice with PBS and fixed in 4% paraformaldehyde for 10 minutes at room temperature. Cells were then permeabilized in 0.1% Triton X-100 in PBS for 15 minutes at room temperature and blocked in blocking buffer (PBS with 10% FBS and 0.1% Triton X-100) for 30 minutes. Cells were incubated with anti-HA antibody (Biolegend, catalog 901501) and anti-Flag antibody (Cell Signaling Technology, catalog 14793S) for 2 hours, followed by Alexa Fluor 488– (Thermo Fisher Scientific, catalog A-11001) and Alexa Fluor 568–conjugated secondary antibodies (Thermo Fisher Scientific, catalog A21069) for 1 hour. Cells were washed with PBS 3 times, mounted on slides using VECTASHIELD mounting medium (Vector Laboratories, catalog H-1000-10), and visualized using an EVOS M5000 cell imaging system (40X objective, Thermo Fisher scientific).

### Statistics.

When indicated, data were analyzed using 2-tailed Student *t* test or ordinary 1-way ANOVA utilizing the GraphPad Prism software (version 9.5.0). Differences were considered significant at *P* < 0.05.

### Study approval.

All patients or their guardians provided written informed consent in accordance with the Declaration of Helsinki under a protocol (NCT01222741) approved by the institutional review board of the National Institute of Allergy and Infectious Diseases, NIH. Blood from healthy donors was obtained under the same approved protocol (NCT01222741).

### Data availability.

Values for all data points in graphs are reported in Supporting Data Values File.

## Author contributions

JS, HSK, RT, and SDR designed the project and wrote the manuscript. RLW, MO, CB, LA, DD, JAK, SMH, and MFS identified the families and were involved in collecting biological and clinical data from the patients. JS, RT, MB, FP, KA, EA, AAGS, and HSK performed experiments, analyzed data, and prepared figures. JEN and JS performed whole-exome sequencing and analyzed data. RT, MB, FP, TAF, HSK, LDN, and SDR analyzed the data and helped in the discussion of ATOs. The order of the co–first authors was determined by the time and effort invested in the project.

## Funding support

This work is the result of NIH funding, in whole or in part, and is subject to the NIH Public Access Policy. Through acceptance of this federal funding, the NIH has been given a right to make the work publicly available in PubMed Central.

## Supplementary Material

Supplemental data

Unedited blot and gel images

Supporting data values

## Version 1 12/22/2025

Electronic publication

## References

[1] Georgopoulos K (1994). The Ikaros gene is required for the development of all lymphoid lineages. Cell.

[2] Heizmann B (2018). The Ikaros family in lymphocyte development. Curr Opin Immunol.

[3] Kuehn HS (2023). Inborn errors of human IKAROS: LOF and GOF variants associated with primary immunodeficiency. Clin Exp Immunol.

[4] Hoshino A (2017). Abnormal hematopoiesis and autoimmunity in human subjects with germline IKZF1 mutations. J Allergy Clin Immunol.

[5] Churchman ML (2018). Germline genetic IKZF1 variation and predisposition to childhood acute lymphoblastic leukemia. Cancer Cell.

[6] Boutboul D (2018). Dominant-negative IKZF1 mutations cause a T, B, and myeloid cell combined immunodeficiency. J Clin Invest.

[7] Ratech H (1997). An analysis of alternatively spliced CD45 mRNA transcripts during T cell maturation in humans. Cell Immunol.

[8] Oberdoerffer S (2008). Regulation of CD45 alternative splicing by heterogeneous ribonucleoprotein, hnRNPLL. Science.

[9] Kuehn HS (2016). Loss of B cells in patients with heterozygous mutations in IKAROS. N Engl J Med.

[10] Hoshino A (2022). Gain-of-function *IKZF1* variants in humans cause immune dysregulation associated with abnormal T/B cell late differentiation. Sci Immunol.

[11] Kuehn HS (2021). Germline IKAROS dimerization haploinsufficiency causes hematologic cytopenias and malignancies. Blood.

[12] Hoshino A (2024). Loss-of-phosphorylation of IKZF1 results in gain-of-function associated with immune dysregulation. J Allergy Clin Immunol.

[13] Klangkalya N (2025). IKAROS protein stability is regulated by its early N-terminal region and C-terminal dimerization domain. Clin Immunol.

[14] Neuber B (2011). Lenalidomide enhances antigen-specific activity and decreases CD45RA expression of T cells from patients with multiple myeloma. J Immunol.

[15] Kronke J (2014). Lenalidomide causes selective degradation of IKZF1 and IKZF3 in multiple myeloma cells. Science.

[16] Perez G (2025). The UCSC Genome Browser database: 2025 update. Nucleic Acids Res.

[17] Thomas RM (2010). Ikaros silences T-bet expression and interferon-gamma production during T helper 2 differentiation. J Biol Chem.

[18] Fujii Y (1992). CD45 isoform expression during T cell development in the thymus. Eur J Immunol.

[19] Fukuhara K (2002). A study on CD45 isoform expression during T-cell development and selection events in the human thymus. Hum Immunol.

[20] Park JE (2020). A cell atlas of human thymic development defines T cell repertoire formation. Science.

[21] Chang X (2016). RNA-binding protein hnRNPLL as a critical regulator of lymphocyte homeostasis and differentiation. Wiley Interdiscip Rev RNA.

[22] Aue G (2018). Activation of Th1 immunity within the tumor microenvironment is associated with clinical response to lenalidomide in chronic lymphocytic leukemia. J Immunol.

[23] Luptakova K (2013). Lenalidomide enhances anti-myeloma cellular immunity. Cancer Immunol Immunother.

[24] Quintana FJ (2012). Aiolos promotes TH17 differentiation by directly silencing Il2 expression. Nat Immunol.

[25] Kuehn HS (2024). Disease-associated AIOLOS variants lead to immune deficiency/dysregulation by haploinsufficiency and redefine AIOLOS functional domains. J Clin Invest.

[26] Yamashita M (2021). A variant in human AIOLOS impairs adaptive immunity by interfering with IKAROS. Nat Immunol.

[27] Kuehn HS (2021). T and B cell abnormalities, pneumocystis pneumonia, and chronic lymphocytic leukemia associated with an AIOLOS defect in patients. J Exp Med.

[28] Sakaguchi S (2000). Regulatory T cells: key controllers of immunologic self-tolerance. Cell.

[29] Park JH (2010). Signaling by intrathymic cytokines, not T cell antigen receptors, specifies CD8 lineage choice and promotes the differentiation of cytotoxic-lineage T cells. Nat Immunol.

[30] Niu N, Qin X (2013). New insights into IL-7 signaling pathways during early and late T cell development. Cell Mol Immunol.

[31] Bosticardo M (2020). Artificial thymic organoids represent a reliable tool to study T-cell differentiation in patients with severe T-cell lymphopenia. Blood Adv.

[32] Ghosh R (2022). FOXI3 haploinsufficiency contributes to low T-cell receptor excision circles and T-cell lymphopenia. J Allergy Clin Immunol.

[33] Speir ML (2021). UCSC Cell Browser: visualize your single-cell data. Bioinformatics.

