# Supplementary material for: IKAROS regulates human T cell phenotype at a thymic and postthymic level
Source: JCI Insight. 2025 Dec 22;10(24):e197359. doi: 10.1172/jci.insight.197359 (PMC12890475; doi:10.1172/jci.insight.197359)
Supplement: Supplemental data [file jciinsight-10-197359-s061.pdf]

Supplemental data

IKAROS regulates human T-cell phenotype at a thymic and post-thymic level

Supplemental Table 1. Immunophenotyping of patients with IKAROS variants.

	A.I.1<br>N159S	B.I.1<br>N159K	B.II.1<br>N159K	B.II.2<br>N159K	C.I.1<br>Y503*	C.II.1<br>Y503*
WBC	***2830***	5120	4450	***3750***	5450	4460
Lymphocytes	48.4/1370	26.6/1360	47.4/2110	28/***1050***	41.1/2240	47.5/2120
CD3	81.3/1114	78.2/1064	78.7/1661	**93.6**/983	77.8/1743	**88.4**/1874
CD3ab	70.8/970	71.9/978	70.1/1479	76.8/806	76.1/1705	81.9/1736
CD3gd	8.9/122	6.3/86	8.7/184	**16.9**/177	1.7/38	6.4/136
CD3/CD4	***18.5/253***	***24.9/339***	***17.2/363***	***21.7/228***	33.2/744	52.2/1107
CD3/CD8	**46.1**/632	**47.5**/646	**53.2/1123**	**62.4**/655	41.6/932	30.1/638
CD4/CD8 ratio	***0.4***	***0.52***	***0.32***	***0.35***	***0.8***	1.73
CD3+/CD4-/CD8-	**16**/219	4.3/58	6.4/135	8.3/87	*1.0/22*	4.7/100
CD3+/CD4+/CD62L+/CD45RA+	73.2/***185***	***2.3/8***	***5.3/19***	***14.9/34***	***6.8/49***	25.3/280
CD3+/CD4+/CD62L+/CD45RA-	11.9/***30***	**79.2**/268	**75.4**/274	**64.8**/*148*	**61.8**/459	**60.0/664**
CD3+/CD4+/CD62L-/CD45RA-	7.6/***19***	18.5/***63***	18.3/***65***	19.9/***45***	30.4/226	14.0/155
CD3+/CD4+/CD62L-/CD45RA+	**7.3**/19	***0.0/0***	1.0/4	0.4/1	1.0/7	0.7/8
CD3+/CD8+/CD62L+/CD45RA+	45.3/286	15.0/97	30.3/340	19.1/125	***11.0***/103	57.4/367
CD3+/CD8+/CD62L+/CD45RA-	*1.2*/***7***	**42.2/273**	28.2/**317**	19.1/125	21.4/**199**	***14.8***/95
CD3+/CD8+/CD62L-/CD45RA-	6.9/44	31.7/205	22.6/252	31.4/206	43.1/**401**	16.5/106
CD3+/CD8+/CD62L-/CD45RA+	46.7/295	11.0/71	18.9/213	30.4/200	24.6/228	11.3/72
CD3+/CD4+/CD45RA+/CD31+	**70.9**/***180***	***0.4/1***	***1.8/7***	9.3/***21***	***4.9/36***	24.7/273
CD20+	***1.3/18***	4.3/***63***	4.8/101	*3.3/35*	4.0/90	3.8/81
CD3-/CD20-/CD16 or CD 56+	**17.4**/238	17.1/233	16.7/352	***2.7/28***	18.6/417	7.7/163

Bold values are high and bold and italic values are low compared to age matched controls. Results are %/absolute count (cells/μl).

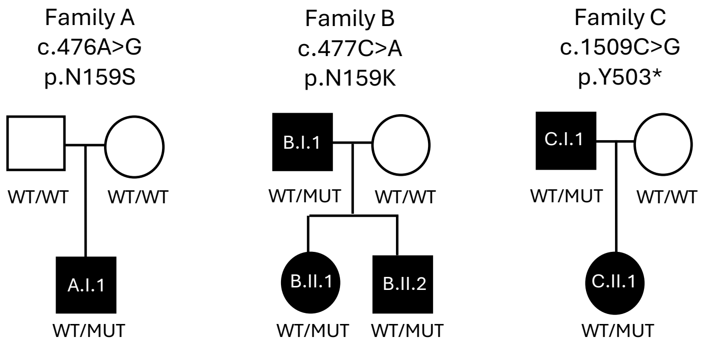

Supplemental Figure 1. Pedigrees of patients with heterozygous *IKZF1* mutations.
Pedigrees of previously unreported patients with IKAROS (IKZF1) variants. Squares indicate males; circles denote females; black filled symbols indicate mutation carriers.

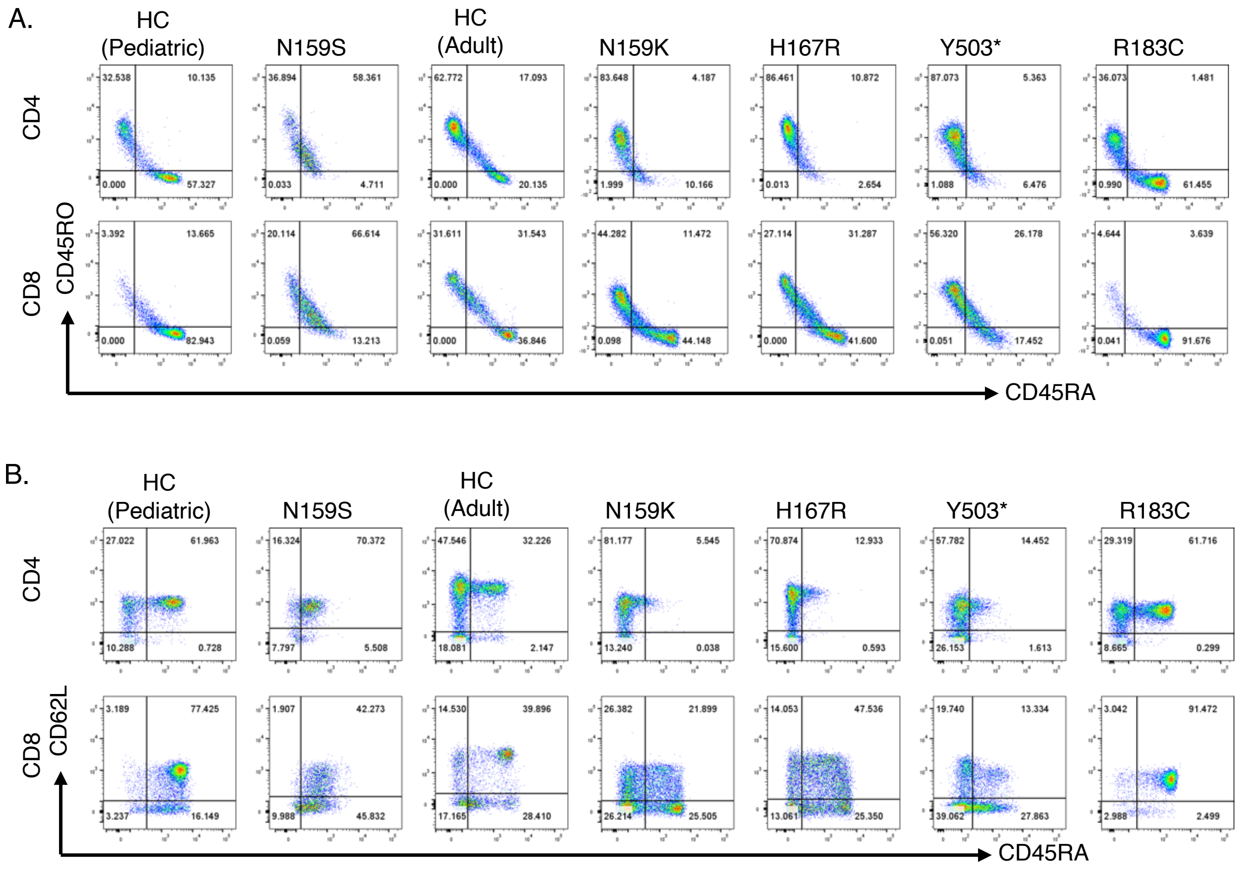

Supplemental Figure 2. Mutations in *IKZF1* result in altered naïve and memory T cell phenotypes. (A and B) T-cell phenotypes in patients with IKAROS variants. N159S is A.I.1, N159K is B.II.2, Y503* is C.I.1., H167R is [C1 in (1)], and R183C is [D3 in (2)]. (A) Naïve (CD45RA$^+$CD45RO$^-$) and memory (CD45RA$^-$CD45RO$^+$) populations and (B) naïve CD4+ T cells (CD45 RA$^+$CD62L$^+$), central memory (CM, CD45RA$^-$CD62L$^+$), effector memory (EM, CD45RA$^-$CD62L$^-$), and TEMRA (CD45RA$^+$CD62L$^-$) populations are shown.

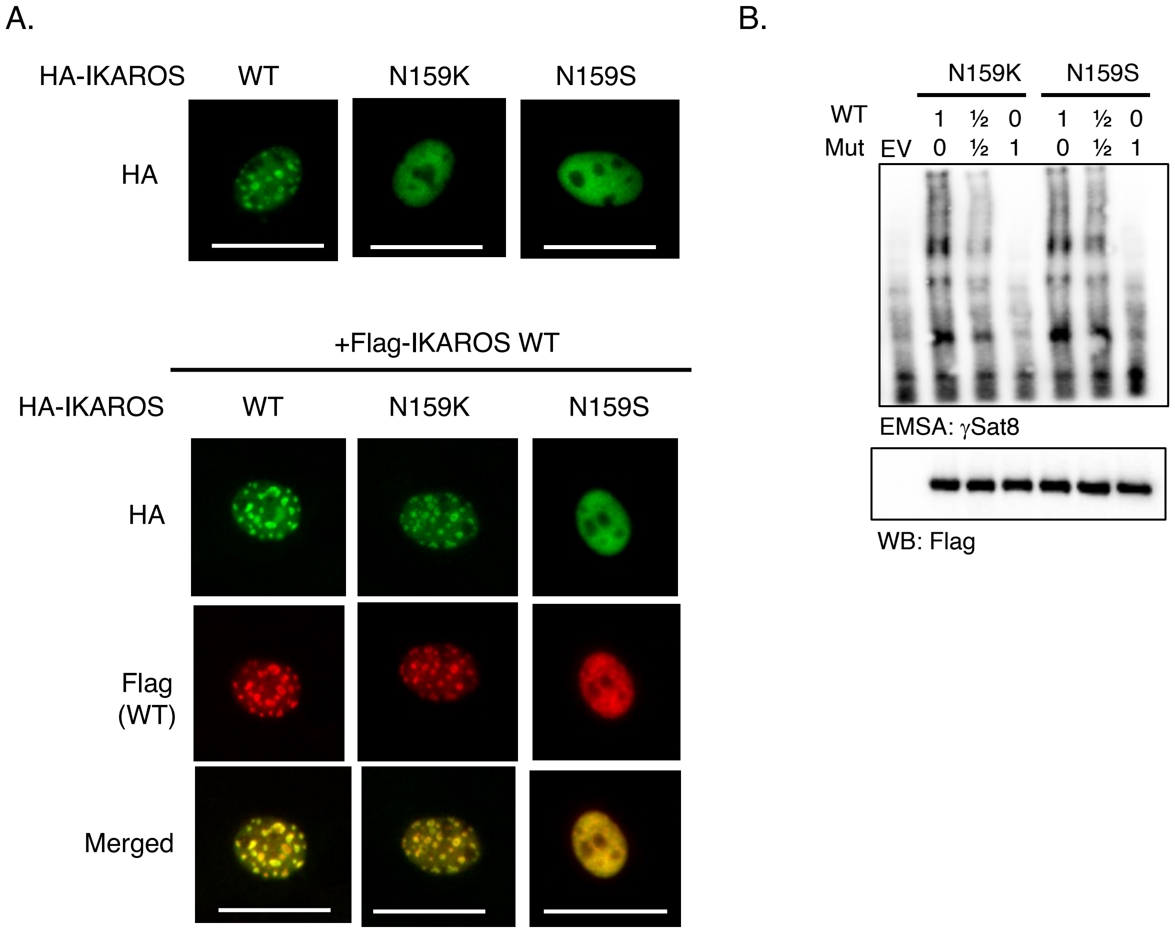

Supplemental Figure 3. Functional tests for the IKAROS variants.
(A) NIH3T3 cells transfected with HA tagged IKAROS WT or the indicated mutants alone (A) or together with Flag-tagged IKAROS WT. Cells were fixed, permeabilized, and stained with HA and Flag antibodies, followed by Alexa 488-conjugated and Alexa 568-conjugated secondary antibodies, respectively. Images were acquired with an EVOS fluorescent microscope (40X objective). The scale bars indicate 25 µm. (B) HEK293T cells transfected with Flag-tagged IKAROS WT and/or the mutants, with the ratios indicated in the figure. After 48 hours, nuclear extracts were prepared and used for the EMSA assay with the γSat8 probe. The Western blot probed with Flag antibodies shows the IKAROS protein expressions used for the EMSA assay. EV indicates empty vector. Data shown are representative of three independent experiments.

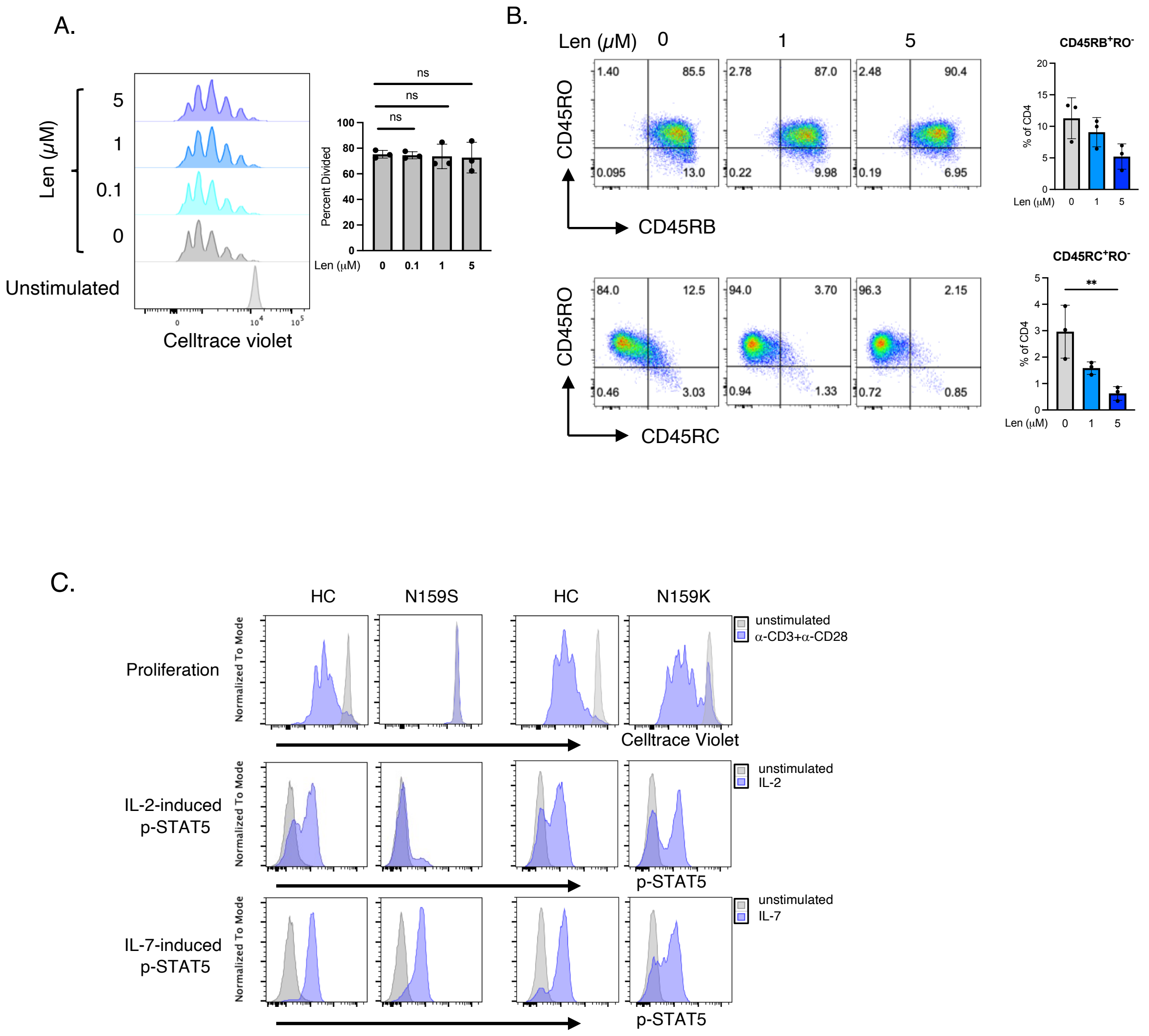

Supplemental Figure 4. Effect of IKAROS on T cell response and T cell phenotypes.
(A) Naïve CD4 T cells were stimulated with Dynabeads T activator-CD3/28 in the presence or absence of the indicated concentrations of lenalidomide. After 4 days of stimulation, T cell proliferation was assessed using Celltrace Violet dilution. The graphs show the mean ± SD from 3 different healthy donor samples. Values indicate the percentage of divided cells (% divided), calculated using FlowJo proliferation platform. Significance was determined using ordinary one-way ANOVA (Dunnett's multiple comparisons test), comparing each group to the Len untreated control. 'ns' indicates not significant. (B) Naïve CD4 T cells were stimulated with Dynabeads T activator-CD3/28 in the presence or absence of the indicated concentrations of lenalidomide for 7 days. CD45RB, CD45RC, and CD45RO expression was measured by flow cytometry. Bar graphs represent mean +/-SD from three independent experiments. Significance was determined using ordinary one-way ANOVA (Tukey's multiple comparisons test), $**p < 0.01$. (C) TCR-induced (soluble anti-CD3 and anti-CD28, 1 μg/ml each) T cell proliferation and STAT5 phosphorylation in response to IL-2 and IL-7 (10 ng/ml) stimulation are shown. CD4 T cells were gated for proliferation and phosphorylation of STAT assay.

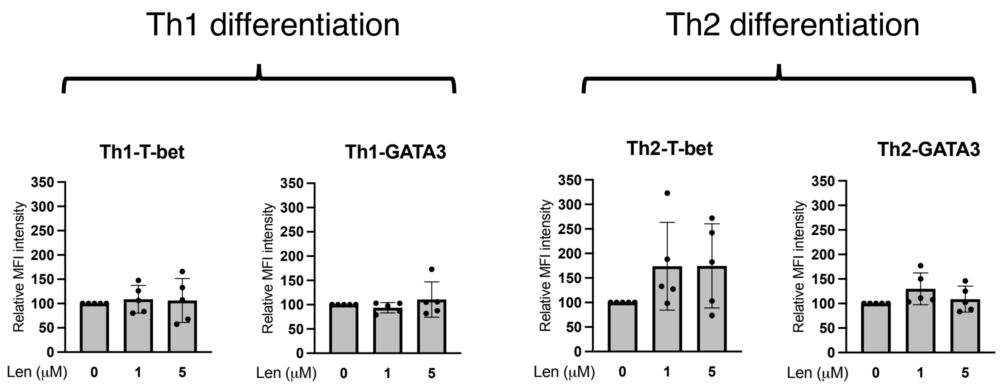

Supplemental Figure 5. Expression of T-bet and GATA3 in Th1 or Th2 differentiation conditions. Enriched naïve $CD4^+$ T cells from healthy controls were cultured with plate bound anti-CD3 and soluble anti-CD28 along with differentiating cytokines (IL-12, IL-2, and anti-IL-4 antibody for Th1 and IL-4, IL-2, and anti-IFN-γ antibody for Th2) in the presence or absence of Lenalidomide (1 or 5 μM). After 7 days of incubation, cells were transferred to plates without anti-CD3 and anti-CD28 stimulation and maintained with IL-2 plus the specific Th-differentiation cytokines for 3 more days in the presence or absence of Lenalidomide. Cells were fixed and permeabilized using the Foxp3 buffer kit, then stained for T-bet and GATA3. The MFI values were normalized to the untreated control, and the graph presents the relative fluorescence intensity under each condition. The graphs show the mean ± SD from 5 different healthy donor samples.

References

1. Kuehn HS, et al. Loss of B Cells in Patients with Heterozygous Mutations in IKAROS. *N Engl J Med.* 2016;374(11):1032-43.
2. Hoshino A, et al. Gain-of-function IKZF1 variants in humans cause immune dysregulation associated with abnormal T/B cell late differentiation. *Sci Immunol.* 2022;7(69):eabi7160.
