# Supplementary figures and images for: IKAROS regulates human T cell phenotype at a thymic and postthymic level
Source: JCI Insight. 2025 Dec 22;10(24):e197359. doi: 10.1172/jci.insight.197359 (PMC12890475; doi:10.1172/jci.insight.197359)

Full unedited gel for Figure 2D

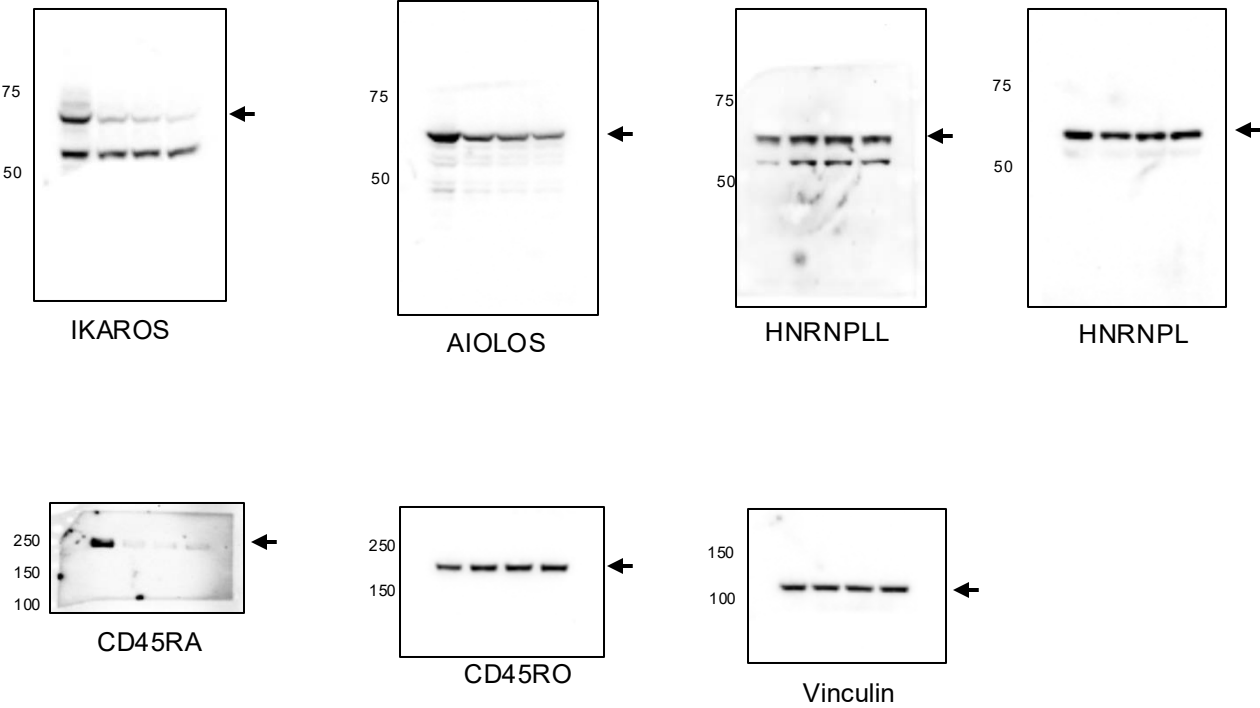

Full unedited gel for Figure 2F

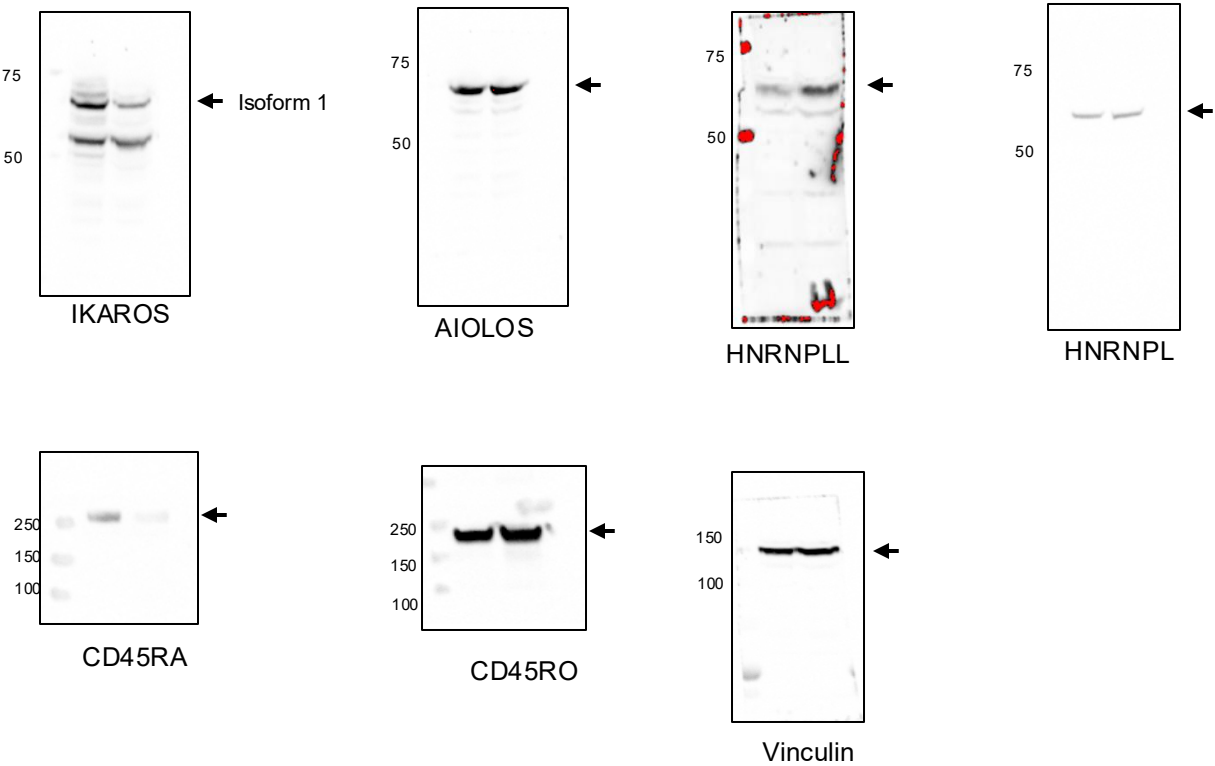

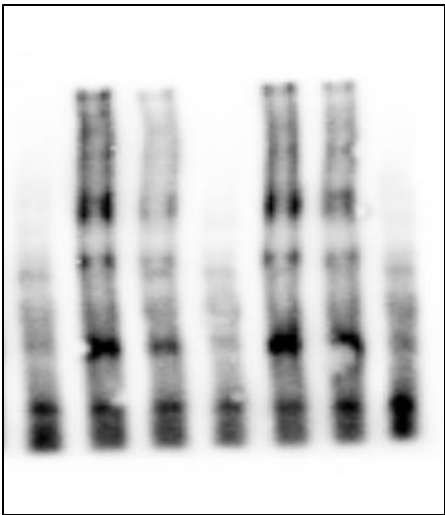

EMSA:  $\gamma$ Sat8

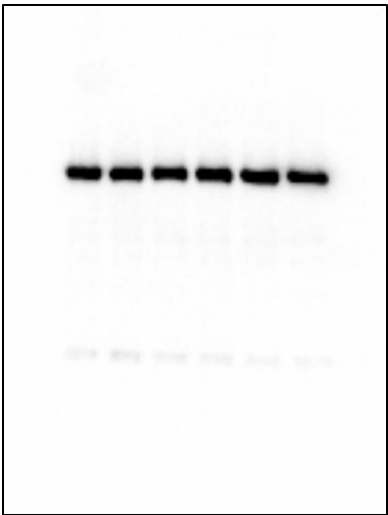

WB: Flag

Supplement: Unedited blot and gel images [file jciinsight-10-197359-s062.pdf]
